# Low Income Amplifies the Negative Relationship Between Nostalgia Proneness and Well-Being

**DOI:** 10.1007/s11482-022-10066-8

**Published:** 2022-05-20

**Authors:** David Benjamin Newman

**Affiliations:** University of California, San Francisco, USA

**Keywords:** Nostalgia, Well-being, Income, Affect, Individual differences

## Abstract

Nostalgia is a sentimental longing for the past that can influence people’s well-being. How this mixed emotion influences well-being may depend on current life circumstances. Nostalgia elicited in negative contexts could be particularly harmful to people’s well-being, whereas nostalgia elicited in positive contexts may not be as detrimental. This hypothesis was tested at the level of individual differences with a nationally representative sample of Americans (*N* = 6,732) who completed measures of nostalgia proneness and several indicators of well-being. Income was measured as an objective indicator of current life circumstances. Results showed that nostalgia proneness was negatively related to well-being, and income was positively related to well-being. Importantly, these relationships were moderated such that the negative relationships between nostalgia and well-being were stronger among members of low income households than among members of high income households. Consistent with the hypothesis, nostalgia proneness was particularly detrimental to well-being under objectively less desirable circumstances. These findings support an emerging body of research that contends that the effect of nostalgia on well-being depends on the context in which nostalgia is elicited.

**Supplementary information:**

The online version contains supplementary material available at 10.1007/s11482-022-10066-8.

Nostalgia is a sentimental longing for the past. It is often characterized as a mixed emotion as it entails the reflection of a positive memory from the past, tinged with a hint of sadness (Batcho, 2013). Similar to most feelings or emotions, nostalgia varies from one situation to the next. Some nostalgic feelings are fairly intense, whereas others are relatively mild. In addition to varying in levels of intensity, nostalgia may vary in valence as well. Although all nostalgic feelings are somewhat bittersweet, some nostalgic feelings may be relatively more sweet than others, whereas other nostalgic feelings might be relatively more bitter than others (Newman et al., 2020). For example, a group of close friends reminiscing about the good old days of their childhood would elicit feelings of nostalgia. Likewise, an individual sitting alone while reflecting on better days would also feel rather nostalgic. Clearly, these nostalgic feelings differ considerably.

Nostalgic feelings can be elicited in a variety of contexts. Some of these contexts are relatively positive, such as interacting with friends or listening to music or singing (Nash, 2012; Newman et al., 2020; Routledge et al., 2011). Other situations that may elicit nostalgia are relatively negative, such as social exclusion, boredom at work, and feelings of meaninglessness (Routledge et al., 2008; Seehusen et al., 2013; van Tilburg et al., 2013; Wildschut et al., 2010). The valence of the situation can influence how positive or negative the nostalgic feeling is, which can subsequently influence well-being in divergent ways (Newman & Sachs, 2020). For example, daily nostalgic feelings were more negatively associated with well-being on days as they felt higher levels of loneliness (Newman & Sachs, 2020). Presumably, nostalgia elicited from lonely states had a more negative effect on well-being than nostalgia elicited in contexts in which people did not feel as lonely.

In the present research, I aim to test this theory at an individual difference level of analysis. In addition to within-person variation in nostalgia (e.g., someone might feel nostalgic in one situation but not during another), nostalgia may vary between persons as well (e.g., some people may feel nostalgic more frequently or intensely than others on average). Individual differences in nostalgia are often termed nostalgia proneness and have been measured in a variety of ways. Well-being was conceptualized as having three aspects: how people evaluate their satisfaction with life (evaluative well-being), how people experience their emotions in daily life (experiential well-being), and how meaningful and purposeful people find their lives (eudaimonic well-being). (See Kahneman 1999; Schwarz & Strack, 1999; Steptoe et al., 2015 for discussions.)

How frequently and/or intensely people feel nostalgic may influence well-being (Muise et al., 2020; Newman et al., 2020). Moreover, the influence of nostalgia proneness on well-being may vary depending on people’s current life situation. One indicator that is often used to assess people’s current situation in a fairly objective manner is income (e.g., Cummins 2000; Deaton, 2008). Money can provide the resources for many needs and benefits, such as access to healthcare and the ability to provide for one’s family. It can also instill a sense of perceived control over life’s uncertainties (Johnson & Krueger, 2006; Kraus et al., 2012; Lachman & Weaver, 1998). When lower income individuals feel a loss of control, they may engage in nostalgic reflections as a means to escape the present reality. This type of bitter nostalgic feeling may lead to lower well-being as it could create a contrast between a better past and the negative present. Moreover, the differing degrees of perceived control that stem from different levels of socioeconomic status can influence the types of emotions people experience (Piff & Moskowitz, 2018; Tong et al., 2021). For instance, members of higher income households report higher levels of positive self-regard emotions like pride, contentment, and confidence, and lower levels negative self-regard emotions like sadness, fear, and shame (Tong et al., 2021). These feelings could influence the types of nostalgic feelings that are typically elicited and could have downstream consequences on their well-being. Of course, it is difficult to measure the different types (i.e., the valence) of nostalgic feelings in person-level measures that require extensive recall, but the theory can nevertheless be tested through the examination of the interactive effects of income on the relationships between nostalgia proneness and well-being.

In light of the prior literature, I hypothesize that the relationship between nostalgia proneness and well-being will be more strongly negative (i.e., more detrimental) at lower levels of income than at higher levels of income. Nostalgic feelings among lower income earners are likely relatively more bitter than sweet compared to nostalgic feelings among higher income earners. Among higher income households, nostalgic feelings may bring to mind positive memories that remind them of the positive aspects of life.

In addition to testing this theory, the secondary goal of the present research is to examine demographic differences in nostalgia proneness. For example, do men and women differ in nostalgia proneness? Do older adults report greater nostalgia than younger adults? Do levels of nostalgia vary by race or ethnicity? Given the advantages of analyzing data from a large, nationally representative sample, it seems valuable to present demographic differences in nostalgia proneness. The latter analyses were relatively exploratory in nature.

## Method

### Participants and Procedure

The sample consisted of participants from the Understanding America Study (https://uasdata.usc.edu), a nationally representative internet panel conducted by the Center for Economic and Social Research (CESR) at the University of Southern California. CESR recruited participants based on various demographics of all household addresses in the US used by Marketing Systems Groups. In this sample, participants without an internet connection were provided with internet-connected tablets to ensure a full coverage of the US population. Participants were given the opportunity to complete multiple questionnaires that have been distributed several times over the year, beginning in 2014. They were financially compensated for each questionnaire completed, and they completed an informed consent prior to participation.

As is fairly common practice in panel data, participants completed some of the measures at different times (e.g., Abakoumkin et al., 2020; Napier & Jost, 2008; Wojcik et al., 2015). The measures of interest for this study were completed primarily during two distributions. Questions about satisfaction with life, positive affect, and negative affect were completed between September 2014 and July 2018. Questions about nostalgia and meaning in life were completed between December 2019 and February 2020. To rule out any potential critiques concerning the fact that some of the well-being variables were measured prior to nostalgia and meaning in life, I also examined the relationships between nostalgia and satisfaction with life with a different measure of satisfaction with life that was administered between February 2021 and April 2021, at least 12 months after the completion of the measures of nostalgia and meaning in life.

Because the analyses focused on nostalgia, demographics completed at the same time as nostalgia were used. Questions about nostalgia, meaning in life, and demographics were answered by 6,732 (*M*_age_ = 49.84, *SD* = 16.16; 58.73% female) participants (see Table 1 for full descriptive statistics). The response rate was 80.68%. Of the 6,732 participants who completed measures of nostalgia, 4,265 participants completed measures of satisfaction with life, positive affect, and negative affect. The response rate for the questionnaire that included measures of satisfaction with life, positive affect, and negative affect was 90.92%. The additional measure of satisfaction with life was completed by 5,798 people who also completed measures of nostalgia. The response rate was 81.00%.

**Table 1:** Participant demographics and descriptive statistics

	*N*	Percentage	Nostalgia *M* (*SD*)
**Gender**			
Male	2778	41.27	3.89 (1.56)
Female	3953	58.73	3.91 (1.61)
**Age**			
18–34 years old	1390	20.67	3.96 (1.59)
35–44 years old	1350	20.07	3.92 (1.61)
45–54 years old	1187	17.65	3.97 (1.62)
55–64 years old	1382	20.55	3.95 (1.57)
65 + years old	1417	21.07	3.73 (1.55)
**Household Income**			
< $30,000	1697	25.25	3.92 (1.70)
$30,000 - $59,999	1760	26.19	3.93 (1.58)
$60,000 - $99,999	1604	23.87	3.90 (1.55)
$100,000 +	1659	24.69	3.86 (1.53)
**Education**			
Low (HS or less)	1562	23.20	3.95 (1.69)
Medium (Some college)	2510	37.28	3.98 (1.58)
High (College degree)	2660	39.51	3.81 (1.53)
**Race/Ethnicity**			
Non-Hispanic White	4288	63.79	3.96 (1.55)
Non-Hispanic Black	528	7.85	3.47 (1.65)
Non-Hispanic Asian	314	4.67	4.13 (1.48)
Hispanic/Latino	1215	18.07	3.86 (1.68)
Other	377	5.61	3.89 (1.61)

Analyses were conducted with as many participants as possible. Because the number of participants who completed nostalgia and meaning in life was larger than those who also completed satisfaction with life and affect, additional analyses were conducted on the smaller subset of participants who completed all measures. Those analyses did not differ substantively from the analyses involving all participants and are presented in Supplemental Materials. The sample size was determined based on the resources available from CESR to conduct the study. Power analyses indicated adequate power (80%) to detect effects as small as *r* = .034. The data are available at https://uasdata.usc.edu/UAS-212 and the analytic code is available at OSF at the following link: https://osf.io/vpdm3/.

## Measures

As is typical in panel studies, several of the constructs were measured with single-items or abbreviated versions of lengthier scales. An advantage of single-items or abbreviated measures is that they minimize participant burden, which is a serious concern in these types of studies. Prior research has shown that single item measures tend to perform just as adequately as their corresponding lengthier measures (Cheung & Lucas, 2014; Gardner et al., 1998; Robins et al., 2001).

Nostalgia proneness was assessed with two items from the Personal Inventory of Nostalgic Experiences (PINE) scale (Newman et al., 2020). The PINE scale has received rigorous psychometric testing, including model fit from confirmatory factor analyses, test-retest reliability, and measurement invariance over time. Before answering the questions about nostalgia, participants were presented with a definition of nostalgia: “According to the Oxford Dictionary, nostalgia is defined as a ‘sentimental longing for the past.’” The two items from the PINE scale were, “How nostalgic do you feel in general?” and “Thinking about your life in general, how much do you feel sentimental for the past?” Responses were reported on a 7-point scale (1 = *do not feel this way at all*, 4 = *feel this way moderately*, 7 = *feel this way very much*; α = 0.87).

Meaning in life was measured with a single item (“My life has a clear sense of purpose or meaning”) that was adapted from the Meaning in Life Questionnaire (Steger et al., 2006). Responses were reported on a 7-point scale (1 = *absolutely untrue*, 7 = *absolutely true*). Questions from the Meaning in Life Questionnaire have demonstrated reasonable levels of reliability over time (Steger & Kashdan, 2007).

Satisfaction with life was measured with a single item (“Overall, how satisfied are you with your life?”). Similarly worded single-item measures have been used to measure satisfaction with life in various panel studies, such as the World Values Survey. Responses were reported on a 10-point scale (1 = *Not at all*, 10 = *Completely*). Global evaluations of satisfaction with life have demonstrated high levels of reliability and stability over time (Anusic & Schimmack, 2016; Schimmack & Oishi, 2005). The additional measure completed in 2021 was the 5-item Satisfaction with Life Scale (α = 0.90; Diener et al., 1985).

To assess positive and negative affect, participants were asked to reflect on their day yesterday. Specifically, they were asked, “What did you do yesterday and how did you feel? To begin, please tell us what time you woke up yesterday.” A series of questions followed that asked about their feelings. “Yesterday, did you feel [adjective]? Would you say:” with response reported on a 10-point scale (1 = *Did not experience this feeling at all*, 10 = *Feeling was extremely strong*). Positive affect was assessed with the following adjectives: happy, enthusiastic, and content; negative affect was assessed with the following adjectives: angry, frustrated, sad, stressed, lonely, worried, and bored. Positive and negative affect demonstrated reasonable reliabilities, αs = 0.91 and 0.86, respectively. Similar to global evaluations of life satisfaction, measures of positive and negative affect have demonstrated reasonably high levels of stability over time (Hudson et al., 2017). Although life satisfaction and affect were measured at a different time than the other measures, the high levels of stability of these global evaluations demonstrate they can be usefully integrated with measures collected more recently. See Nezlek (2021) and Abakoumkin et al., (2020) for similar procedures.

## Results

### Preliminary analyses

Before testing the primary hypothesis, I present descriptive statistics for two purposes. The first is to document demographic differences in nostalgia proneness as this is the first nationally representative sample of Americans to complete a nostalgia proneness measure^1^. The second purpose is to provide convergent validity of our single-item and abbreviated scales.

Descriptive statistics and demographic differences in nostalgia proneness are documented in Table 1. Males (*M* = 3.89, *SD* = 1.56) and females (*M* = 3.91, *SD* = 1.61) did not differ in how nostalgic they felt in general, *t*(6729) = 0.54, 95% CI [-0.06, 0.10], *p* = .587. Nostalgia was negatively related to age, *r*(6724) = − 0.04, 95% CI [-0.07, − 0.02], *p* < .001, although this effect appeared to be driven by the fact that adults older than 65 (*M* = 3.73, *SD* = 1.55) reported lower nostalgia than adults younger than 65 (*M* = 3.95, *SD* = 1.60). Nostalgia was not significantly related to age among those younger than 65, *r*(5307) = − 0.00, 95% CI [-0.03, 0.03], *p* = .982. Nostalgia was not significantly related to income, *r*(6718) = − 0.02, 95% CI [-0.05, 0.00], *p* = .072. Nostalgia was negatively related to education, *r*(6730) = − 0.04, 95% CI [-0.06, − 0.02], *p* = .001, and this effect was driven by the fact that the most highly educated people (those with at least a Bachelor’s degree; *M* = 3.81, *SD* = 1.53) report lower levels of nostalgia than those without a Bachelor’s degree (*M* = 3.97, *SD* = 1.62), *t*(6730) = -4.02, 95% CI [-0.24, − 0.08], *p* < .001. In terms of race and ethnicity, Non-Hispanic Asian participants reported the highest levels of nostalgia (*M* = 4.13, *SD* = 1.48), and Non-Hispanic Black participants reported the lowest levels of nostalgia (*M* = 3.47, *SD* = 1.65), with Non-Hispanic White participants (*M* = 3.96, *SD* = 1.55), other (*M* = 3.89, *SD* = 1.61), and Hispanic/Latino participants (*M* = 3.86, *SD* = 1.68) following in between.

To provide support for convergent validity, correlations between nostalgia, well-being, and all continuous demographic variables (age, income, and education) are presented in Table 2. Nostalgia proneness was negatively related to satisfaction with life, meaning in life, and positive affect and was positively related to negative affect, consistent with correlations documented by Newman et al., (2020, Study 2) that used longer versions of each measure.^2^ The single-item measure of meaning in life was positively related to satisfaction with life and positive affect and was negatively related to negative affect, consistent with studies that have used longer versions of these measures (e.g., Newman et al., 2018; Steger et al., 2006). Meaning in life was also positively related to age and income, consistent with prior research (e.g., Steger et al., 2009; Ward & King, 2016). Thus, our abbreviated measures demonstrated adequate convergence validity.

**Table 2:** Correlation table for nostalgia, well-being, and continuous demographic variables

	Nostalgia	Meaning in life	Satisfaction with life	Positive affect	Negative affect	Age	Income
Nostalgia							
Meaning in life	− 0.10						
Satisfaction with life	− 0.14	0.33					
Positive affect	− 0.14	0.27	0.65				
Negative affect	0.18	− 0.20	− 0.47	− 0.62			
Age	− 0.04	0.04	0.10	0.06	− 0.18		
Income	− 0.02	0.12	0.21	0.15	− 0.09	0.03	
Education	− 0.04	0.06	0.07	0.08	− 0.03	0.04	0.43

Note: All correlations were significant at *p* < .01 except for the correlations between nostalgia and income (*p* = .072) and between education and negative affect (*p* = .084).

## Primary analyses

The primary analyses addressed an interaction effect of nostalgia and income on well-being. Is the relationship between nostalgia proneness and well-being moderated by income? To answer this question, I examined two main effects of nostalgia on well-being and income on well-being before testing the interaction. I used ordinary least squares regression in R and standardized all continuous predictors and outcomes. The analytic syntax is available at OSF: https://osf.io/vpdm3/.

In separate models, each well-being variable was regressed on nostalgia and age, gender, education, income, and race/ethnicity. Nostalgia was negatively related to satisfaction with life, *B* = − 0.13, 95% CI [-0.16, − 0.11], *t* = -9.10, *p* < .001, meaning in life, *B* = − 0.09, 95% CI [-0.11, − 0.06], *t* = -7.22, *p* < .001, positive affect, *B* = − 0.12, 95% CI [-0.15, − 0.09], *t* = -8.15, *p* < .001, and was positively related to negative affect, *B* = 0.17, 95% CI [0.14, 0.20], *t* = 11.57, *p* < .001^3^. Generalizing the findings from Newman et al., (2020), nostalgia was negatively related to well-being controlling for demographic variables. Next, in separate models, each well-being variable was regressed on income and the same set of demographic controls. Income was positively related to satisfaction with life, *B* = 0.24, 95% CI [0.21, 0.27], *t* = 14.22, *p* < .001, meaning in life, *B* = 0.13, 95% CI [0.11, 0.16], *t* = 9.81 *p* < .001, positive affect, *B* = 0.16, 95% CI [0.13, 0.20], *t* = 9.47, *p* < .001, and negatively related to negative affect *B* = − 0.11, 95% CI [-0.15, − 0.08], *t* = -6.52, *p* < .001.

**Fig. 1:**
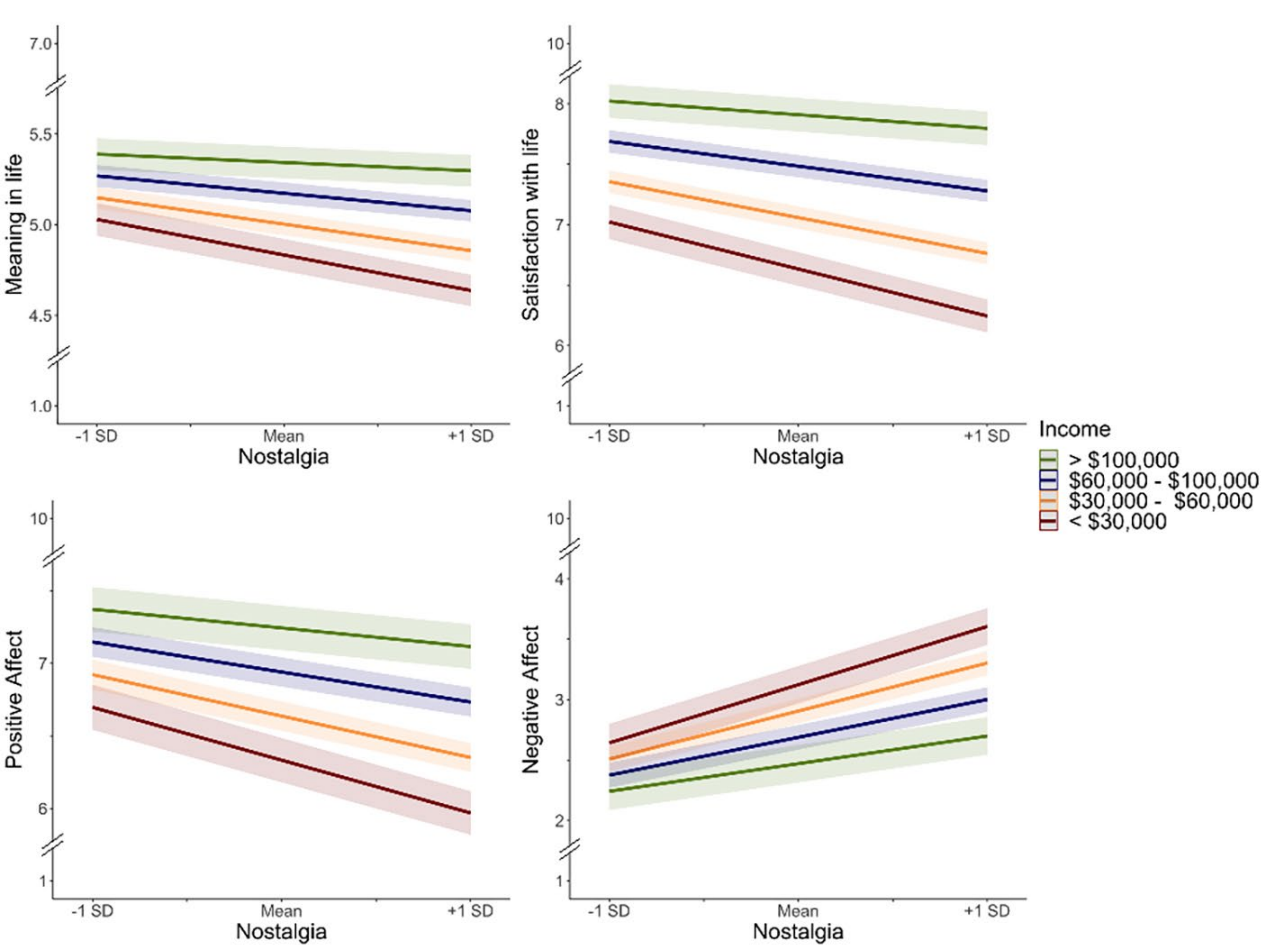
Interactive effects of nostalgia and income on well-being. Note: Dashed lines indicate the figures are zoomed in to more easily display the direction of the relationships. The negative relationships between nostalgia and well-being are stronger among lower income households than among higher income households

Critically, interaction effects were tested by including nostalgia, income, and an interaction term along with demographic controls as predictors. Interaction terms were significant for satisfaction with life, *B* = 0.15, 95% CI [0.06, 0.23], *t* = 3.35, *p* < .001, meaning in life, *B* = 0.12, 95% CI [0.05, 0.18], *t* = 3.30, *p* < .001, positive affect, *B* = 0.10, 95% CI [0.02, 0.19], *t* = 2.35, *p* = .019, and negative affect, *B* = − 0.12, 95% CI [-0.20, − 0.03], *t* = -2.68, *p* = .007^4^. The relationship between nostalgia and well-being was moderated by income such that the negative relationship between nostalgia and well-being was stronger among lower income households than among higher income households, as depicted in Fig. 1. When viewed alternatively, the positive relationship between income and well-being was stronger among those high in nostalgia proneness than among those low in nostalgia proneness.

The interaction analysis that used the Satisfaction with Life Scale (Diener et al., 1985) completed after nostalgia replicated those with the measure of satisfaction with life completed prior to nostalgia, *B* = 0.11, 95% CI [0.02, 0.20], *t* = 2.51, *p* = .012. (See Supplemental Materials for additional details.) Thus, the analyses were robust across different measures of well-being completed at different times.

## Discussion

The present study served exploratory and confirmatory research agendas: documenting demographic differences in nostalgia proneness and testing a theory that argues that the relationship between nostalgia proneness and well-being is context dependent. As this dataset is the first nationally representative sample of Americans to assess nostalgia proneness, documenting demographic differences is important for generating hypotheses for future research. There were no gender differences in nostalgia proneness. Those over age 65 reported lower nostalgia than those younger than 65. Highly educated individuals reported lower nostalgia than the less well educated, but income was not associated with nostalgia. Among the different races and ethnicities represented in the dataset, Non-Hispanic Asian participants reported the highest levels of nostalgia while Non-Hispanic Black participants reported the lowest levels.

The key purpose of this research was to test a theory about contextual effects of nostalgia proneness on well-being (e.g., Newman & Sachs 2020). Under favorable conditions, nostalgia may be relatively more sweet than bitter and may subsequently predict greater well-being. In contrast, when life is not going well, nostalgic feelings may be characterized as relatively more bitter than sweet and could predict lower well-being. In a similar vein, the within-person relationships between daily nostalgic states and well-being were more strongly negative when people felt lonely than when they did not feel as lonely (Newman & Sachs, 2020).

The present findings provide additional support for this theory by demonstrating that the negative relationships between nostalgia proneness and well-being were significantly stronger among lower income households. Presumably, nostalgic feelings among lower income households are elicited in less favorable situations or circumstances compared to higher income households. The contextual effects of income levels can subsequently predict well-being.

Before discussing implications and future directions, it is important to note that the findings and theory appear at first glance to contrast with research that has suggested that nostalgia can be used as a buffer against negative experiences (Sedikides et al., 2015). For instance, following the death of a loved one, individual differences in nostalgia predicted less intrusive thoughts over a one month period (Reid et al., 2021). In a different study among Syrian refugees, recalling their most nostalgic feeling increased certain positive emotions, self-continuity, meaning in life, self-esteem, and social connectedness compared to reflections of an ordinary experience (Wildschut et al., 2019). These findings suggest that nostalgia can serve a palliative function by alleviating the negative effects of dire situations.

To reconcile the apparent discrepancies between prior research and the present findings, it is important to consider methodological and theoretical factors. The studies that have documented palliative effects of nostalgia proneness (i.e., measured as an individual difference) have primarily relied on the Southampton Nostalgia Scale (SNS; Barrett et al., 2010) whereas the present study relied on the Personal Inventory of Nostalgic Experiences (PINE; Newman et al., 2020) Scale. The SNS confounds nostalgia proneness with nostalgia seeking or valuing nostalgia, a component closely linked to approach motivation (Newman et al., 2020). In contrast, the items from the PINE Scale are motivationally neutral. This suggests that the act of valuing or seeking nostalgia could serve as a buffer against negative experiences, whereas the tendency to simply feel nostalgic may not. In other studies, participants are specifically asked to engage in the recollection of a very nostalgic experience, which tends to have positive effects (Sedikides et al., 2015). Similar to clinical trials or interventions, the reflection of a specific, highly nostalgic feeling may have certain benefits that can alleviate negative effects. Thus, whereas prior research has demonstrated that nostalgia may have beneficial effects in certain negative circumstances, the present study shows that nostalgia proneness measured in a motivationally neutral manner is particularly negatively related to well-being. The present study does not negate or contradict prior research. Rather, the present study addresses a unique question.

In addition to the contextual effects of nostalgia on well-being, the present study augments our understanding of the potential effects of income on well-being. The positive relationships between income and various well-being measures replicate previous research that has measured these constructs at the level of individual differences (e.g., Diener et al., 2010; Kahneman & Deaton, 2010; Ward & King, 2016). The present findings also show that not only does income relate positively to well-being, but the relationship is stronger among people high in nostalgia proneness. That is, income seems to more strongly predict well-being among people who tend to feel highly nostalgic than among people who feel less nostalgic.

Interpreted in an alternative manner, the results could suggest that income serves as a buffer against the negative effects of nostalgia. Higher levels of income are associated with a sense of freedom, control, and choice (Kraus et al., 2012), which means that income could allow people to engage in nostalgic thoughts when it is convenient and somewhat beneficial. Lower income individuals tend to engage with their environment because they need to manage threats and external constraints (Kraus et al., 2009), and this could mean they are forced to engage in nostalgic feelings during inopportune moments when it could be particularly detrimental to their well-being. Additionally, income could provide the resources to alleviate the pains associated with nostalgia. For example, the sadness that accompanies a nostalgic feeling for a childhood friend who lives far away could be offset among those who have the financial resources to afford a trip to visit the childhood friend, thus improving one’s well-being. Admittedly speculative, this possibility requires studies designed to test such processes. Nevertheless, the current research provides a nuanced understanding of how the effect of income on well-being varies as a function of nostalgia proneness.

The present research has implications beyond nostalgia for mixed emotions more broadly. Recent research has documented a range of consequences of mixed emotional experiences. For instance, mixed emotions predict a higher likelihood of engaging in preventative health behaviors during the Covid-19 pandemic (Oh & Tong, 2021) and can attenuate declines in physical health (Hershfield et al., 2013). Mixed emotions have also predicted greater eudaimonic well-being (Berrios et al., 2018) and improved psychological well-being (Adler & Hershfield, 2012). In contrast, other studies have shown that mixed emotions predict lower well-being roughly ten years later (Oh, 2022). People who experience high levels of mixed emotions in daily life also tend to report high levels of neuroticism (Barford et al., 2020), which is negatively associated with well-being. Although the present results do not clarify these inconsistent findings, they could point to potential explanations for these discrepancies by emphasizing the importance of moderating factors. Just as income moderated the relationship between nostalgia proneness and well-being, other moderating factors could point to reasons why mixed emotions may be beneficial in certain contexts and harmful in others.

## Limitations and future directions

A few limitations and directions for future research are worth noting. Similar to panel studies with measures assessed at different times, some of the well-being measures in the present study were administered prior to nostalgia, whereas others were measured at the same time or after nostalgia. Outcome measures are ideally assessed after predictors; this limitation was addressed by analyses that showed that the pattern of results remained the same across all well-being outcomes measured at different times. More importantly though, given the cross-sectional and correlational nature of the data, causal claims cannot be made. Although the data are consistent with a model that involves causal processes, the correlations could be interpreted differently. It is possible that well-being might cause people to feel more or less nostalgic over time, and well-being and nostalgia could potentially influence people’s income. As income is difficult to manipulate (as are individual differences), longitudinal studies would be necessary to examine lagged processes over time. Longitudinal studies would also be valuable in teasing apart cohort effects or factors pertaining specifically to major world events, such as the Covid-19 pandemic. Because the interaction effects were similar for both satisfaction with life measures which were measured before and after the onset of the Covid-19 pandemic, it suggests the findings may be robust across such contexts, but additional research would be needed. Additionally, the exact mechanisms explaining why nostalgia elicited among members of lower income households is negatively related to well-being are difficult to discern in cross-sectional data. Nostalgia elicited in negative situations may make the negative aspects associated with nostalgic feelings quite salient or they may remind people that their current situation is not as rosy as their remembered past (Iyer & Jetten, 2011; Osborn et al., 2020). Future research is necessary is examine the precise mechanisms.

As is common practice in panel studies, some of the measures were assessed with one or two items. Although abbreviated scales often converge with results from lengthier corresponding scales as was documented in these data, single item scales may not capture the complex facets of a construct. For instance, recent work has suggested that meaning in life is a higher order factor that consists of three facets: purpose, coherence, and significance (George & Park, 2016; Martela & Steger, 2016). Future research could examine how nostalgia and income influence specific facets of well-being in differential ways. Relatedly, the present study included two items that assessed personal nostalgia. Recent work has shown that personal nostalgia and collective nostalgia may have divergent effects on attitudes and prejudice (Behler et al., 2021; Wohl et al., 2020). It would be fruitful to examine the effects of income and different forms of nostalgia (e.g., collective nostalgia, anticipatory nostalgia; Batcho 2020; Batcho & Shikh, 2016) on well-being in future research.

## Summary

In conclusion, nostalgia proneness is negatively related to well-being, but this relationship is context sensitive. The strength of this association may vary depending on people’s current life circumstances. The negative relationship between nostalgia proneness and well-being is amplified among members of lower income households. Although correlational, these findings are consistent with a process model that shows that nostalgia elicited in negative situations can have particularly detrimental effects on well-being.

## Open Practices Statement

The data are available at https://uasdata.usc.edu/UAS-212 to the public and the analytic syntax is available at the OSF (https://osf.io/vpdm3/) for the purpose of verification of the results presented in this manuscript. The analyses were not formally preregistered.

## Electronic supplementary material

Below is the link to the electronic supplementary material.

Supplementary Material 1

## Data Availability

Data are available at https://uasdata.usc.edu/UAS-212 and the syntax is available at: https://osf.io/vpdm3/

## Footnotes

1 The Longitudinal Internet studies for the Social Sciences (LISS), a longitudinal panel study with a probability sample of Dutch participants, includes the Southampton Nostalgia Scale, a measure of nostalgia that combines nostalgia proneness with nostalgia seeking/valuing (Abakoumkin et al., 2020).

2 The negative correlations between nostalgia proneness and positive activated and positive deactivated affect reported by Newman et al., (2020, Study 2) were not significantly different from zero but were trending in the negative direction.

3 A common analysis in the mixed emotions literature is to control for the effects of positive and negative affect. After controlling for positive and negative affect when satisfaction and meaning were regressed on nostalgia and demographic controls, nostalgia was still negatively related to satisfaction and meaning. This suggests that the predictive role of nostalgia on well-being cannot be solely attributed to one particular valence. See Supplemental Materials for details.

4 Similar to before, additional models controlled for the effects of positive and negative affect in predicting satisfaction and meaning in life. The interaction terms remained significant or marginally significant. Details are provided in Supplemental Materials.

## References

[CR1] Abakoumkin, G., Wildschut, T., & Sedikides, C. (2020). Nostalgia proneness and the collective self. *Frontiers in Psychology*, *11*. 10.3389/fpsyg.2020.570621 10.3389/fpsyg.2020.570621 PMC7649288 33192861

[CR2] Adler JM, Hershfield HE (2012). Mixed emotional experience is associated with and precedes improvements in psychological well-being. PLOS ONE.

[CR3] Anusic I, Schimmack U (2016). Stability and change of personality traits, self-esteem, and well-being: Introducing the meta-analytic stability and change model of retest correlations. Journal of Personality and Social Psychology.

[CR4] Barford KA, Koval P, Kuppens P, Smillie LD (2020). When good feelings turn mixed: Affective dynamics and big five trait predictors of mixed emotions in daily life. European Journal of Personality.

[CR5] Barrett FS, Grimm KJ, Robins RW, Wildschut T, Sedikides C, Janata P (2010). Music-evoked nostalgia: Affect, memory, and personality. Emotion.

[CR6] Batcho KI (2013). Nostalgia: The bittersweet history of a psychological concept. History of Psychology.

[CR7] Batcho KI, Shikh S (2016). Anticipatory nostalgia: Missing the present before it’s gone. Personality and Individual Differences.

[CR8] Batcho, K. I. (2020). When nostalgia tilts to sad: Anticipatory and personal nostalgia. *Frontiers in Psychology*, *11*. 10.3389/fpsyg.2020.01186 10.3389/fpsyg.2020.01186 PMC7274075 32547466

[CR9] Behler AMC, Cairo A, Green JD, Hall C (2021). Making America great again? National nostalgia’s effect on outgroup perceptions. Frontiers in Psychology.

[CR10] Berrios R, Totterdell P, Kellett S (2018). When feeling mixed can be meaningful: The relation between mixed emotions and eudaimonic well-being. Journal of Happiness Studies.

[CR11] Cheung F, Lucas RE (2014). Assessing the validity of single-item life satisfaction measures: Results from three large samples. Quality of Life Research.

[CR12] Cummins RA (2000). Objective and subjective quality of life: An interactive model. Social Indicators Research.

[CR13] Deaton A (2008). Income, health, and well-being around the world: Evidence from the Gallup World Poll. Journal of Economic Perspectives.

[CR14] Diener E, Ng W, Harter J, Arora R (2010). Wealth and happiness across the world: Material prosperity predicts life evaluation, whereas psychosocial prosperity predicts positive feeling. Journal of Personality and Social Psychology.

[CR15] Gardner DG, Cummings LL, Dunham RB, Pierce JL (1998). Single-item versus multiple-item measurement scales: An empirical comparison. Educational and Psychological Measurement.

[CR16] George LS, Park CL (2016). Meaning in life as comprehension, purpose, and mattering: Toward integration and new research questions. Review of General Psychology.

[CR17] Hershfield HE, Scheibe S, Sims TL, Carstensen LL (2013). When feeling bad can be good: Mixed emotions benefit physical health across adulthood. Social Psychological and Personality Science.

[CR18] Hudson NW, Lucas RE, Donnellan MB (2017). Day-to-day affect is surprisingly stable: A 2-year longitudinal study of well-being. Social Psychological and Personality Science.

[CR19] Iyer A, Jetten J (2011). What’s left behind: Identity continuity moderates the effect of nostalgia on well-being and life choices. Journal of Personality and Social Psychology.

[CR20] Johnson W, Krueger RF (2006). How money buys happiness: Genetic and environmental processes linking finances and life satisfaction. Journal of Personality and Social Psychology.

[CR21] Kahneman, D. (1999). Objective happiness. In D. Kahneman, E. Diener, & N. Schwarz (Eds.), *Well-being: Foundations of hedonic psychology* (pp. 3–25). Russell Sage Foundation

[CR22] Kahneman, D., & Deaton, A. (2010). High income improves evaluation of life but not emotional well-being. *Proceedings of the National Academy of Sciences*, *107*(38), 16489–16493. 10.1073/pnas.1011492107 10.1073/pnas.1011492107 PMC2944762 20823223

[CR23] Kraus MW, Piff PK, Keltner D (2009). Social class, sense of control, and social explanation. Journal of Personality and Social Psychology.

[CR24] Kraus MW, Piff PK, Mendoza-Denton R, Rheinschmidt ML, Keltner D (2012). Social class, solipsism, and contextualism: How the rich are different from the poor. Psychological Review.

[CR25] Lachman ME, Weaver SL (1998). The sense of control as a moderator of social class differences in health and well-being. Journal of Personality and Social Psychology.

[CR26] Martela F, Steger MF (2016). The three meanings of meaning in life: Distinguishing coherence, purpose, and significance. The Journal of Positive Psychology.

[CR27] Muise A, Kim JJ, Debrot A, Impett EA, MacDonald G (2020). Sexual nostalgia as a response to unmet sexual and relational needs: The role of attachment avoidance. Personality and Social Psychology Bulletin.

[CR28] Napier JL, Jost JT (2008). Why are conservatives happier than liberals?. Psychological Science.

[CR29] Nash JE (2012). Ringing the chord: Sentimentality and nostalgia among male singers. Journal of Contemporary Ethnography.

[CR30] Newman DB, Nezlek JB, Thrash TM (2018). The dynamics of searching for meaning and presence of meaning in daily life. Journal of Personality.

[CR31] Newman, D. B., & Sachs, M. E. (2020). The negative interactive effects of nostalgia and loneliness on affect in daily life. *Frontiers in Psychology*, *11*. 10.3389/fpsyg.2020.02185 10.3389/fpsyg.2020.02185 PMC7492671 32982886

[CR32] Newman DB, Sachs ME, Stone AA, Schwarz N (2020). Nostalgia and well-being in daily life: An ecological validity perspective. Journal of Personality and Social Psychology.

[CR33] Nezlek JB (2021). Relationships Among Belief in God, Well-Being, and Social Capital in the 2020 European and World Values Surveys: Distinguishing Interpersonal and Ideological Prosociality. Journal of Religion and Health.

[CR34] Oh VYS, Tong EMW (2021). Mixed emotions, but not positive or negative emotions, facilitate legitimate virus-prevention behaviors and eudaimonic outcomes in the emergence of the COVID-19 crisis. Affective Science.

[CR35] Oh VYS (2022). Torn between valences: Mixed emotions predict poorer psychological well-being and job burnout. Journal of Happiness Studies.

[CR36] Osborn H, Markman KD, Howell JL (2020). Nostalgia and temporal self-appraisal: Divergent evaluations of past and present selves. Self and Identity.

[CR37] Piff PK, Moskowitz JP (2018). Wealth, poverty, and happiness: Social class is differentially associated with positive emotions. Emotion.

[CR38] Reid CA, Green JD, Short SD, Willis KD, Moloney JM, Collison EA, Gramling S (2021). The past as a resource for the bereaved: Nostalgia predicts declines in distress. Cognition and Emotion.

[CR39] Robins RW, Hendin HM, Trzesniewski KH (2001). Measuring global self-esteem: Construct validation of a single-item measure and the Rosenberg Self-Esteem Scale. Personality and Social Psychology Bulletin.

[CR40] Routledge C, Arndt J, Sedikides C, Wildschut T (2008). A blast from the past: The terror management function of nostalgia. Journal of Experimental Social Psychology.

[CR41] Routledge C, Arndt J, Wildschut T, Sedikides C, Hart CM, Juhl J, Schlotz W (2011). The past makes the present meaningful: Nostalgia as an existential resource. Journal of Personality and Social Psychology.

[CR42] Schimmack U, Oishi S (2005). The influence of chronically and temporarily accessible information on life satisfaction judgments. Journal of Personality and Social Psychology.

[CR43] Schwarz, N., & Strack, F. (1999). Reports of subjective well-being: Judgmental processes and their methodological implications. In D. Kahneman, E. Diener, & N. Scshwarz (Eds.), *Well-being: Foundations of hedonic psychology* (pp. 61–84). Russell Sage Foundation

[CR44] Sedikides C, Wildschut T, Routledge C, Arndt J, Hepper EG, Zhou X (2015). To nostalgize: Mixing memory with affect and desire. Advances in Experimental Social Psychology.

[CR45] Seehusen J, Cordaro F, Wildschut T, Sedikides C, Routledge C, Blackhart GC, Vingerhoets AJJM (2013). Individual differences in nostalgia proneness: The integrating role of the need to belong. Personality and Individual Differences.

[CR46] Steger MF, Frazier P, Oishi S, Kaler M (2006). The meaning in life questionnaire: Assessing the presence of and search for meaning in life. Journal of Counseling Psychology.

[CR47] Steger MF, Kashdan TB (2007). Stability and specificity of meaning in life and life satisfaction over one year. Journal of Happiness Studies.

[CR48] Steger MF, Oishi S, Kashdan TB (2009). Meaning in life across the life span: Levels and correlates of meaning in life from emerging adulthood to older adulthood. The Journal of Positive Psychology.

[CR49] Steptoe A, Deaton A, Stone AA (2015). Subjective wellbeing, health, and ageing. The Lancet.

[CR50] Tong, E. M. W., Reddish, P., Oh, V. Y. S., Ng, W., Sasaki, E., Chin, E. D. A., & Diener, E. (2021). Income robustly predicts self-regard emotions. *Emotion*, No Pagination Specified-No Pagination Specified. 10.1037/emo0000933 10.1037/emo0000933 33661663

[CR51] van Tilburg WAP, Igou ER, Sedikides C (2013). In search of meaningfulness: Nostalgia as an antidote to boredom. Emotion.

[CR52] Ward SJ, King LA (2016). Poor but happy? Income, happiness, and experienced and expected meaning in life. Social Psychological and Personality Science.

[CR53] Wildschut T, Sedikides C, Routledge C, Arndt J, Cordaro F (2010). Nostalgia as a repository of social connectedness: The role of attachment-related avoidance. Journal of Personality and Social Psychology.

[CR54] Wildschut T, Sedikides C, Alowidy D (2019). Hanin: Nostalgia among Syrian refugees. European Journal of Social Psychology.

[CR55] Wohl MJA, Stefaniak A, Smeekes A (2020). Longing is in the memory of the beholder: Collective nostalgia content determines the method members will support to make their group great again. Journal of Experimental Social Psychology.

[CR56] Wojcik SP, Hovasapian A, Graham J, Motyl M, Ditto PH (2015). Conservatives report, but liberals display, greater happiness. Science.

