# Supplementary material for: Low Income Amplifies the Negative Relationship Between Nostalgia Proneness and Well-Being
Source: Appl Res Qual Life. 2022 May 20;17(6):3311–26. doi: 10.1007/s11482-022-10066-8 (PMC9121087; doi:10.1007/s11482-022-10066-8)
Supplement: Supplementary file 1 — Supplementary Material 1 [file 11482_2022_10066_MOESM1_ESM.docx]

**Supplemental Materials**

The primary analyses were conducted with as many participants as possible for each model. Because the number of participants who completed nostalgia and meaning in life was larger than those who also completed satisfaction with life and affect, additional analyses were conducted on the smaller subset of participants who completed all measures.

In these models, nostalgia was negatively related to satisfaction with life, *B* = -.13, 95% CI [-.16, -.10], *t* = -9.00, *p* < .001, meaning in life, *B* = -.09, 95% CI [-.12, -.06], *t* = -5.75, *p* < .001, positive affect, *B* = -.12, 95% CI [-.15, -.09], *t* = -8.03, *p* < .001, and was positively related to negative affect, *B* = .17, 95% CI [.14, .20], *t* = 11.55, *p* < .001.

Next, in separate models, each well-being variable was regressed on income and the same set of demographic controls. Income was positively related to satisfaction with life, *B* = .24, 95% CI [.21, .27], *t* = 14.21, *p* < .001, meaning in life, *B* = .13, 95% CI [.09, .16], *t* = 7.37 *p* < .001, positive affect, *B* = .16, 95% CI [.13, .20], *t* = 9.46, *p* < .001, and negatively related to negative affect *B* = -.11, 95% CI [-.15, -.08], *t* = -6.52, *p* < .001.

Interaction effects were tested by including nostalgia, income, and an interaction term along with demographic controls as predictors. Interaction terms were significant for satisfaction with life, *B* = .14, 95% CI [.06, .23], *t* = 3.27, *p* = .001, meaning in life, *B* = .15, 95% CI [.07, .24], *t* = 3.43, *p* < .001, positive affect, *B* = .10, 95% CI [.02, .19], *t* = 2.38, *p* = .018, and negative affect, *B* = -.11, 95% CI [-.20, -.03], *t* = -2.59, *p* = .010. These results were functionally equivalent to those reported in the manuscript.

In addition to the primary analyses conducted on a smaller subset of the sample, I examined the same analyses with a different measure of satisfaction with life as mentioned in the main text. The single item measure of satisfaction with life was administered before the nostalgia and meaning in life questions. Thus, although prior research has demonstrated considerable stability of these measures over time, some may wonder why satisfaction with life (a dependent variable) is measured prior to the independent variable. To address this limitation, I therefore examined the same set of analyses with a different measure of satisfaction with life that was assessed after nostalgia. To compare the results of these analyses with the prior analyses, I conducted these analyses with participants who completed both measures of satisfaction with life.

First, the two measures of satisfaction with life were positively related, *r*(3739) = .52, *p* < .001, demonstrating a reasonable amount of consistency over time. Zero order correlations showed that satisfaction with life was positively related to meaning in life, *r*(3735) = .40, *p* < .001, positive affect, *r*(3729) = .39, *p* < .001, age, *r*(3736) = .01, *p* = .477, income, *r*(3734) = .32, *p* < .001, education, *r*(3739) = .15, *p* < .001, and was negatively related to nostalgia, *r*(3739) = -.14, *p* < .001, and negative affect, *r*(3729) = -.29, *p* < .001. Testing the first main effect, nostalgia proneness was negatively related to satisfaction with life after controlling for demographics, *B* = -.15, 95% CI [-.18, -.12], *t* = -9.39, *p* < .001. The other main effect showed that income was positively related to satisfaction with life after controlling for demographics, *B* = .31, 95% CI [.27, .34], *t* = 17.47, *p* < .001. Finally, as reported in the main text, the interaction term of nostalgia proneness and income was significant, *B* = .11, 95% CI [.02, .20], *t* = 2.51, *p* = .012. As depicted in Supplemental Figure 1, the relationship between nostalgia proneness and satisfaction with life was more strongly negative among lower income households than among higher income households.

Supplemental Figure 1. Interaction effect of nostalgia and income on satisfaction with life.


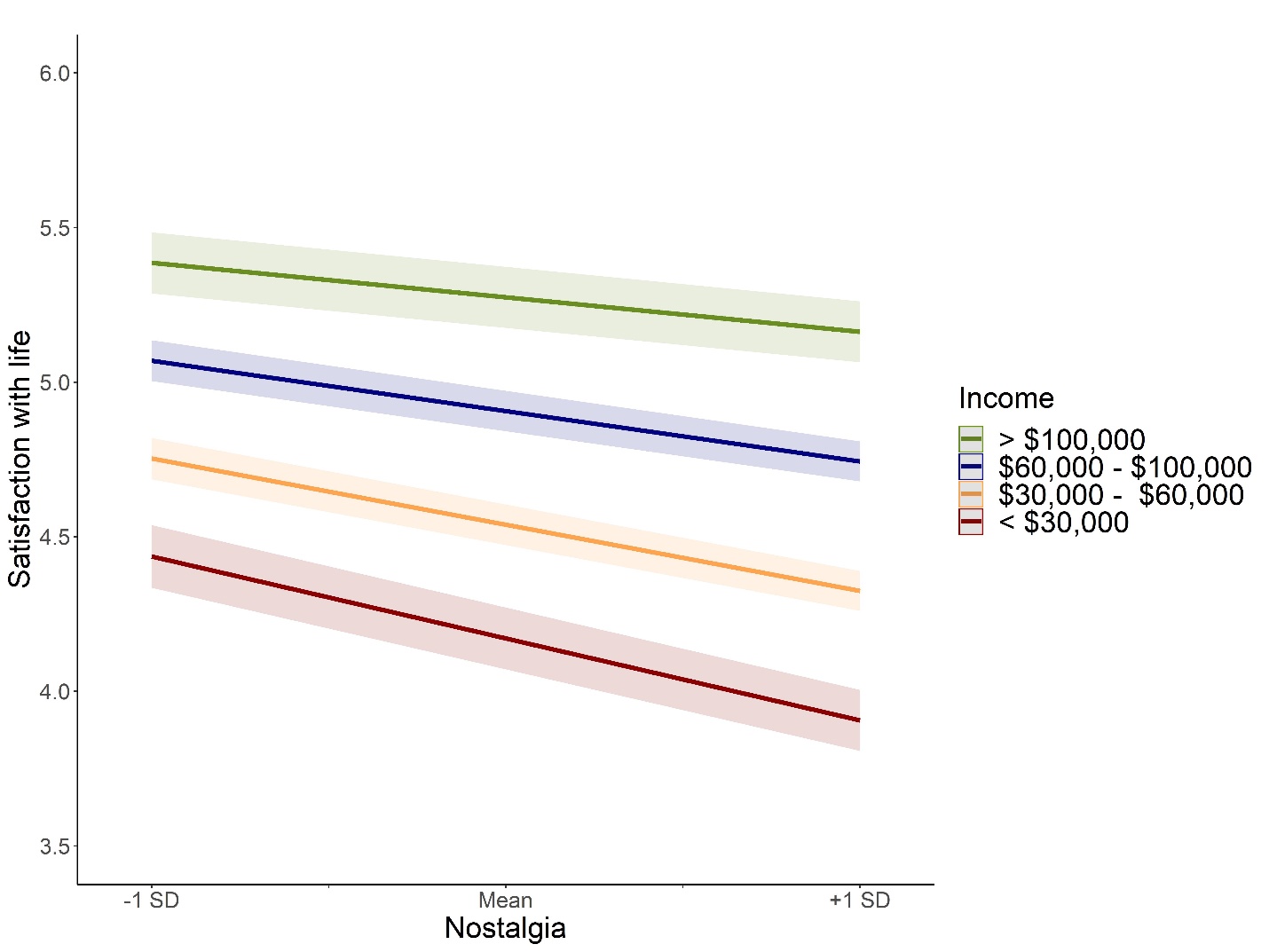


Finally, as mentioned in Footnotes 3 and 4, a common approach in the mixed emotions literature is to control for the effects of positive and negative affect when examining the relationship between a mixed emotion and an outcome. Therefore, I controlled for positive and negative affect in the main effect analyses in which nostalgia predicted meaning in life and satisfaction with life. Demographic variables were also included as controls. Nostalgia was negatively related to meaning in life, *B* = -.05, 95% CI [-.08, -.02], *t* = -3.55, *p* < .001, the single-item satisfaction with life measure, *B* = -.05, 95% CI [-.07, -.03], *t* = -4.31, *p* < .001, and the 5-item satisfaction with life measure, *B* = -.10, 95% CI [-.13, -.07], *t* = -6.86, *p* < .001. Thus, the negative relationships between nostalgia and satisfaction with life and meaning in life cannot be fully explained by broad measures positive or negative valence.

Additionally, positive and negative affect were included as control predictors in the interaction models. The interaction terms in models predicting meaning in life, *B* = .13, 95% CI [.04, .21], *t* = 2.91, *p* = .004, and the single-item satisfaction with life measure, *B* = .07, 95% CI [.01, .14], *t* = 2.20, *p* = .028, remained significant. The interaction term in the model predicting the 5-item satisfaction with life measure, *B* = .08, 95% CI [-.01, .16], *t* = 1.79, *p* = .074, became marginally significant. Thus, the interactive effects of nostalgia with income on well-being cannot be solely attributed to general levels of positive or negative valence.
